# Strangulated Hernia—Herniotomy—Recovery

**Published:** 1888-06

**Authors:** W. J. Love

**Affiliations:** Wacoochee, Ala.


### STRANGULATED HERNIA—HERNIOTOMY RECOVERY.

BY W. J. LOVE, M. D., WACOOCHEE, ALA.

On the 15th of February, 1888, I was called to see T. B., male, 45 years of age, stout, fleshy and plethoric. I found him suffering with a large scrotal hernia of the right side. The tumor was as large as a cocoanut, hard, nodulated and very tender to the touch. He gave the following history:

He had been ruptured about five years; had worn a truss most of the time. The bowel had been down several times before; three times had to have a physician and chloroform, reduction being accomplished with much pain and difficulty; he had been troubled for several days previous to present attack with diarrhœa, and the bowel had come down about three hours before while patient was lifting a heavy weight; had vomited a great deal at first, and was still suffering with nausea and occasional vomiting of fecal matter. There had been several discharges from bowels, and was now a continual desire to go to stool, with severe pain in right iliac region and severe tormina. All this, he said, was very different from anything he had felt in previous attacks. His pulse was 110, temperature 100° F., skin covered with large drops of perspiration; the slightest manipulation at the tumor increased the pain and fecal vomiting. I feared at once a case of strangulated gut, but decided to try the usual methods of taxis; chloroform was administered until complete anæsthesia was produced, but all efforts at reduction proved a failure, and only seemed to increase the severity of all the symptoms; and after several vain attempts I gave it up and decided that nothing but an operation would save my patient, and that every moment's delay increased his danger. I gave him, hypodermically,

Morphia Sulph., . . . . . . . . . . . . . . gr. ¼

Atropia Sulph, . . . . . . . . . . . . . . gr. $\frac{1}{120}$

This produced several hours' sleep, and on the 16th, assisted by Drs. McCoy, Palmer and J. M. Love, the operation was performed. The patient was put under chloroform, put on table, shoulders slightly raised and knees flexed on a pillow. The integument over the parts was shaved, and the scrotum and lower abdomen were washed with five per cent. solution carbolic acid; hands of those present, and all the instruments to be used, went through a like process. I then made an incision along the inguinal canal, from the internal to just below the external abdominal ring; in this incision all the soft parts were severed consecutively down to the constricted portion, each layer of tissue being divided to the full extent of the wound, and several vessels were twisted. The sac now being exposed, I felt for the stricture, and found it just outside the neck, and after considerable difficulty and several failures, I finally succeeded in passing a Levis director under the band from below upwards; the stricture was then divided with a hernia knife, making the incision something more than (¼) one-fourth of an inch in length; this exposed several old bands of adhesions that were with difficulty divided. I then attempted sac-reduction, but without success; the sac was bound down with so many old bands of adhesive lymph that I finally gave it up, and opened it by making a free incision, being careful not to wound its contents. There was a quick rush of bloody-looking fluid, followed by a fold of intestine, bulging up through the incision. This being examined showed considerable congestion, and was spotted with ecchymosis; some spots were quite black, and in places the bowel was granular from exuded lymph, but nowhere was the vitality of the bowel entirely destroyed. The sac contained several folds of the ilium and a small quantity of omentum; this was all very gently, but thoroughly, sponged off with solution of boracic acid, and gently returned into the abdominal cavity, the contents of sac passing through the internal ring very easily. The wound was now carefully cleansed with the boracic acid solution and closed with carbolized silk sutures. The parts were dusted with powdered iodoform, being first well dried. Over this were placed two folds of iodoform gauze, a firm pad placed on top of that, and secured with a spica bandage, and patient was put to bed in good condition. I saw him two hours later, found his pulse 120, temperature 101° F., respiration 26, some pain in lower abdominal regions, with desire to go to stool.

I introduced into rectum suppository containing one-half grain sulph. morphia; this quieted the pain, also the desire to go to stool, in an hour, but patient continued restless, complained of thirst; throat and mouth were dry. I gave, hypodermically,

Morphia Sulph, . . . . . . . . . . . . . gr. $\frac{1}{6}$

Atropia Sulph., . . . . . . . . . . . . . gr. $\frac{1}{120}$

Ordered turpentine emulsion given every three hours, containing ten drops turpentine and one drop carbolic acid at a dose. On visiting him the next day, I found his temperature 99° F., pulse 94, no thirst and no pain. He had slept several hours during the night, and said he was hungry. I directed diet of oatmeal, tea and crackers, only a small quantity to be given at a time. In twenty-four hours after operation, the patient had a copious discharge from his bowels without any pain or nausea, his bladder also acting normally. After this his pulse and temperature rapidly returned to normal. On the second day I examined the wound with a view of using a water dressing, but on removing the folds of gauze, the iodoform that was dusted over the wound when it was dressed looked dry and in good condition; not the slightest moisture was present. I only put on fresh gauze. On the fifth day I again examined the wound and removed the sutures. It seemed almost entirely well; only a small blue line where the incision was made. There was no tenderness on pressure, and the general condition of the patient was all that could be desired. On the seventh day I allowed him to sit up, and on the tenth day he walked about his room. A well-fitting truss being put on before resuming the upright position, the pressure of the truss caused no pain or uncomfortable feeling of any kind. The patient is now going about, and says he feels as well as he did before the operation was performed.

The points of special interest in this case are the rapidity with which the inflamed intestine assumed a normal condition, the manner in which the wound healed (by first intention) and the power of boracic acid as a disinfectant.

